# Effects of Cactus Polysaccharide on Pasting, Rheology, Structural Properties, In Vitro Digestibility, and Freeze–Thaw Stability of Rice Starch

**DOI:** 10.3390/foods13152420

**Published:** 2024-07-30

**Authors:** Yahui Zhu, Chuang Dong, Fumin Chi, Xuedong Gu, Lei Liu, Lin Yang

**Affiliations:** 1 College of Food Science, Tibet Agriculture & Animal Husbandry University, Nyingchi 860000, China; zhuyahui@xza.edu.cn (Y.Z.);; 2 College of Food Science, The Provincial and Ministerial Co-Founded Collaborative Innovation Center for R & D in Tibet Characteristic Agricultural and Animal Husbandry Resources, Nyingchi 860000, China

**Keywords:** cactus polysaccharide, rice starch, pasting properties, rheology, in vitro digestibility

## Abstract

This study combined rice starch (RS) with cactus polysaccharide (CP) at different composites (0.6%, 1.2%, 1.8%, 2.4%, and 3.0%, *w*/*w*), and analyzed the variations in the complex gelatinization properties, rheological properties, thermal properties, structural properties, digestibility, and freeze–thaw stability. As a result, the pasting parameters (*p* < 0.05) and storage modulus (G′) together with the loss modulus (G″) decreased as the CP concentration increased; meanwhile, the RS and the CP–RS gels were pseudoplastic fluids. As revealed by differential scanning calorimetry (DSC), incorporating CP into the starch elevated the starch gelatinization temperature while decreasing gelatinization enthalpy, revealing that CP effectively retarded long-term retrogradation in RS. The gel microstructure and crystallization type altered after adding CP. Typically, CP inclusion could enhance the proportion of resistant starch and slowly digestible starch (SDS), thereby slowing RS hydrolysis. Concurrently, adding CP promoted the RS freeze–thaw stability. These findings could potentially aid in the innovation of CP-based food products.

## 1. Introduction

In Asia, rice accounts for the typical staple food due to its eating quality, textural, hypoallergenic, and ease-of-preparation features [1,2]. Starch, a major component in rice, exerts a crucial effect on the human diet. However, native starches are associated with some demerits, including low shear resistance, freeze–thaw and thermal stability, easy digestion, and retrogradation. Such undesirable traits significantly restrict the application of native starch in the food field [3]. Gelatinized RS includes over 95% rapidly digestible starch (RDS), which can be easily digested and absorbed, leading to a high glycemic response. This phenomenon does not correspond to the modern concept of nutrition and health [4].

Nowadays, non-starch polysaccharides (NSPs) are extensively utilized as functional additives in starch-based systems to improve the nutritional and sensory properties of starchy foods [5,6]. However, owing to the differences in starch crystal structure and starch suspension charge, NSPs have an erratic influence on the pasting properties of diverse starches [7]. For example, the corn starch pasting curve increased after adding tamarind polysaccharide [8]. The overall pasting viscosity as well as the trough viscosity (TV), peak viscosity (PV), and final viscosity (FV) of sweet potato starch increased because of the interaction between the starch and xanthan gum or mesona chinensis polysaccharide (MCP), respectively [9]. Furthermore, wheat starch had reduced TV, PV, and FV after adding MCP and konjac gum, respectively [10,11]. Meanwhile, the setback viscosity (SV) value of starch was reduced because of sodium alginate and pectin [12,13]. These results can be interpreted as representing the competition of NSPs against starch for free water, which increases leaching of amylose molecules and effective concentrations of starch granules and inhibits starch pasting [14]. The rheological characteristics of starch, such as shear-thinning behavior [8], flow behavior index (n) [15], viscosity [16], storage modulus (G′), and loss modulus (G″) are altered via NSPs [17]. Native starch structural degradation induced by NSPs and their physical obstructive effects are two contributing factors that modulate starch digestibility [18].

Opuntia dillenii, a member of the cactus family, thrives in tropical and subtropical climates and is typically found in regions with arid or semi-desert conditions. This plant species is cultivated in various regions of China, including Guangxi, Jiangxi, and Fujian. It is highly versatile, being used for food, ornamental, and medicinal applications [19]. The polysaccharide from Opuntia dillenii (ODP) is primarily sourced from the cactus’s fruit and stem, and its composition includes arabinose, fructose, glucose, and rhamnose [20]. As a key constituent of cactus’s bioactive profile, ODP has numerous biological functions, including antioxidation [21], hypolipidaemia [22], antidiabetes [23], and antitumor properties [24]. Recently, many studies have been conducted to examine how ODP-based edible coatings affect fruit and vegetable preservation [25,26]. Nevertheless, there is a scarcity of scientific literature exploring the interaction between CP and starch properties.

Consequently, the present work focused on investigating how varying CP doses affected RS gelatinization properties, rheological properties, structural properties, thermal properties, digestibility, and freeze–thaw stability. This research may offer foundational insights for harnessing NSPs to improve starch-based foods with regard to their processing traits and storage stability.

## 2. Materials and Methods

### 2.1. Materials and Reagents

Fresh cactus cladodes (*Opuntia ficus-indica* (Linn.) Mill.) were collected from Chawalong Township, Chayu County, Tibet, in September 2023. The samples were transported back to the laboratory in a vehicle-mounted refrigerator (CL-43DM, Midea Group, Hefei, China) and processed immediately. RS (14.3% amylose) and the glucose detection kit were obtained from Shanghai Yuanye Co., Ltd. (Shanghai, China). Anhydrous ethanol, sodium acetate buffer solution, α-amylase, and amyloglucosidase were purchased from China Pharmaceutical Chemicals Ltd. (Shanghai, China).

### 2.2. CP Preparation

The cactus thorns and peels were removed to collect the pulp, the pulp was freeze-dried and ground into powder (through 100 mesh). Then, the powder was dissolved into water at a 1:30 (*w*/*v*) solid–liquid ratio and stirred continuously. The extraction time was 4 h and the temperature was 80 °C in a water bath. After extraction, the product was centrifuged (JXN-26, Beckman Coulter, Inc., Brea, CA, USA) at 10,000 rpm for 20 min. Proteins were removed via the utilization of Sevag reagent. Absolute ethanol was added into this solution and the solution was stored at 4 °C in a refrigerator for 12 h (final ethanol concentration: 80%). After precipitation, the precipitate was collected for dialysis and stored at 4 °C in a refrigerator (dialysis membrane 3500 Da, 3 d). The dialysate was freeze-dried into cactus polysaccharide.

### 2.3. Preparation of CP-RS Composites

We synthesized CP–RS (0.6%, 1.2%, 1.8%, 2.4% and 3.0%, *w*/*w*) composites together with the gelatinized solutions in line with specific protocols after some modification [27], as shown in Table 1.

### 2.4. Pasting Properties

CP–RS pasting properties were analyzed with a Rapid Visco Analyzer (RVA) (3-D, Newport Scientific, Sydney, Australia) according to Kong et al.’s description [28]. The CP–RS was weighed to precisely 2.86 g and subsequently, the powdered CP–RS mixtures were dispersed in aluminum crucibles with 25.14 mL distilled water. After thorough preliminary stirring with a small plastic propeller, the aluminum crucible was locked into the rotating spindle of the RVA, and the spindle cap was pressed to initiate the measurement. The RVA testing protocol was configured such that the stirring speed was set at 960 rpm for a brief 10 s, then the speed was reduced to 160 rpm for a consistent rotation throughout the test. Firstly, the sample was heated to 50 °C and held for 1 min. Then, the temperature was raised from 50 to 95 °C at a constant rate of 12 °C/min. After holding at 95 °C for 2.0 min, the temperature was decreased to 50 °C at the same scanning rate and held at 50 °C for 2.5 min. The pasting curves and parameters were obtained using the TCW (Thermal Cycle for Windows) 3.0 software, which was compatible with RVA.

### 2.5. Rheological Measurements

Rheological measurements of CP–RS gels acquired through RVA analysis were measured with a rheometer (DHR-2, TA Instruments, New Castle, DE, USA) using a parallel plate (diameter, 40 mm; gap, 1 mm) [29]. Samples were added onto the parallel plate prior to 1 min of equilibration at 25 °C before testing. We ascertained the linear viscoelastic interval of the sample gel (Supplementary Figure S1) via a strain sweep examination (0.01–10%) at 25 °C and 1 Hz. The strain parameter was selected as 1% for subsequent experiments. All measurements were carried out three times in parallel.

#### 2.5.1. Dynamic Rheological Properties

This study derived dynamic moduli storage modulus (G′), loss modulus (G″), and loss factor (tan δ = G″/G′) from oscillatory rheological tests within the specified frequency spectrum of 0.1~10 Hz. The regression between G′ (G″) and angular frequency (ω) of the CP–RS gels was calculated according to the following formulas:

G′ = k′ × ω^n′^ (1)

G″ = k″ × ω^n″^ (2)

where k′ (Pa·s^n^) and k″ (Pa·s^n^) indicate the power law constants, n′ and n″ indicate the frequency indexes, and ω indicates the angular frequency (rad/s). 

#### 2.5.2. Steady Shear Rheology

We programmed the parallel plate under steady shear mode to obtain an increasing shear rate within 0–300 s^−1^ (upward flow curve), and then decreased it from 300 to 0 s^−1^ (downward flow curve). Additionally, the Herschel–Bulkley model expressed by Formula (3) was adopted for data fitting:

τ = τ_0_ + Kγ^n^ (3)

in which τ represents shear stress (Pa), τ_0_ indicates yield stress (Pa), γ stands for shear rate (s^−1^), n demonstrates flow behavior index, and K is indicative of the consistency coefficient (Pa∙s^n^). 

### 2.6. Thermal Property Analysis

CP–RS gelatinization was analyzed via DSC (DSC-214, Netzsch, Selb, Germany) following Tao et al.’s approach [30] after certain modifications. Later, a 3.0 mg sample was blended into distilled water (9.0 µL) and put into an aluminum pan, followed by 12 h of balancing at 4 °C after sealing. We heated the aluminum pan under nitrogen protection (40 mL/min) from 20 to 130 °C at 10 °C/min, with the empty aluminum pan being the control. 

### 2.7. Scanning Electron Microscope (SEM)

We determined gel microstructure as described by Wang et al. [31] after mild modifications. The gels obtained from RVA were subjected to pre-freezing at −80 °C and freeze drying (FreeZone, Labconco, Kansas city, MO, USA). Their microstructures were observed with SEM (SU3500, Hitachi, Kyoto, Japan) at a 20 kV acceleration voltage. Photographs were captured at 200× magnification.

### 2.8. X-ray Diffraction (XRD)

XRD (XRD-6100, Shimazu, Kyoto, Japan) was adopted for analyzing crystalline characteristics following the method described by Takahashi and Fujita. Ref. [32] with a minor modification. RVA was conducted for obtaining samples. A freeze drier was adopted for gel drying, whereas a 300-mesh sieve was utilized to sort powder according to size. The scanning angles were 5°–60° (2θ), while scanning speed was set at 10°/min. 

### 2.9. Fourier Transforms Infrared Spectroscopy (FT-IR)

After grinding samples with KBr within the agate mortar at a 1:50 ratio, the mixture was ground to fine powder and pressed to a sheet [33]. CP–RS infrared spectra were obtained within 4000–400 cm^−1^ with FT-IR (Nicolet 5700, Thermo Scientific, Waltham, MA, USA). 

### 2.10. In Vitro Digestion

Digestion properties were investigated according to Yin et al. [34]. Samples, as described in Section 2.4, were pulverized into a fine powder (100 mesh, 50 mg, dry weight) and then mixed with 50 mL acetate buffer solution (0.2 M, pH 6.0). This mixture was allowed to stand at 37 °C within the water bath for a 20 min period before introducing 5 mL of a combined enzyme solution (2900 U/mL α-amylase and 150 U/mL amyloglucosidase). This reaction mixture was kept at 37 °C within the water bath throughout the process. At predetermined time points (0, 20, and 120 min), 0.1 mL aliquots of the digested fluid were extracted. Subsequently, anhydrous ethanol (0.5 mL) was used for enzyme deactivation, followed by 10 min centrifugation at 4000 rpm. Later, 0.1 mL supernatant was added into 3.0 mL GOPDO reagents to react at 50 °C for a 20 min period. Then, absorbance values were read at 510 nm. RDS, SDS, and resistant starch levels were determined using Formulas (4)–(6).

Indicate rapidly digestible starch% = (G_20_ − G_0_) × 0.9 × 100/TS (4)

Slowly digestible starch% = (G_120_ − G_20_) × 0.9 × 100/TS (5)

Resistant starch% = (TS − RDS − SDS) × 100/TS (6)

in which, G_0_, G_20_, and G_120_ represent glucose levels at 0, 20, and 120 min, respectively, and TS represents starch total dry weight. 

### 2.11. Freeze–Thaw Stability

The freeze–thaw stability of CP–RS was tested following the method of Zhai et al. [35]. The powder samples (2.0 g) with different proportions were mixed with distilled water (20 mL) and were then subjected to 30 min continuous stirring within a boiling water bath. The cooled solution (20.0 g, 30 °C) was added into a preweighed centrifuge tube (50 mL) and the total weight was recorded. The freeze–thaw cycle operation was as follows: 22 h of sample freezing at −18 °C, 2 h of thawing at 30 °C, and 20 min centrifugation at 4000 rpm following the freeze–thaw cycle for 1, 3, 5, and 7 times. Supernatants were poured off before weighing. The process was conducted five times for assessing samples’ freeze–thaw stability. The syneresis rate was defined using Formula (7):

Syneresis (%) = (M_2_ − M_3_)/(M_2_ − M_1_) × 100% (7)

where M_1_ (g), M_2_ (g), M_3_ (g) represent the weights of the centrifugal tube, starch paste and centrifuge tube, and starch paste and centrifuge tube excluding supernatant, respectively.

### 2.12. Statistical Analysis

SPSS 20.0 (Inc., Armonk, NY, USA) was adopted for data analysis. Differences between means were examined with Duncan’s multiple range tests. *p* < 0.05 represented significant difference, and the rheology parameters were defined using Trios (Version Number: 5.1.1.46572, New Castle, DE, USA).

## 3. Results and Discussion

### 3.1. Pasting Characteristics

**Figure 1:**
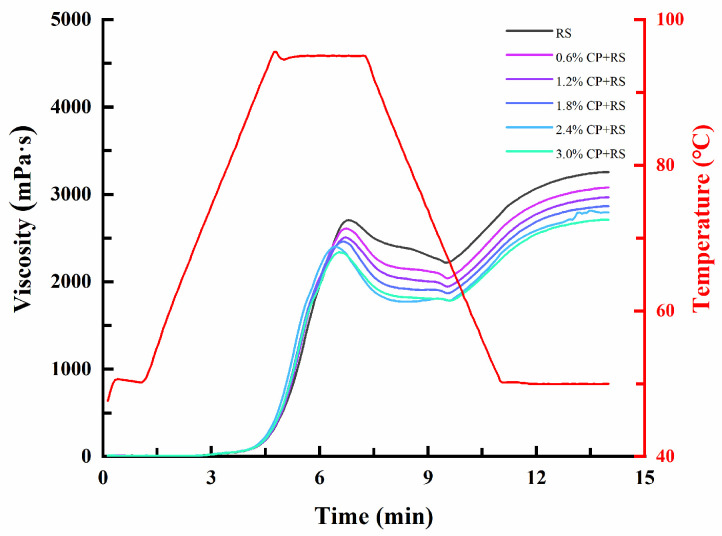
RVA pasting profiles of RS and CP–RS gels of diverse CP concentrations.

RVA was conducted to characterize RS gelatinization behavior with CP (0.6, 1.2, 1.8, 2.4, and 3.0%, *w*/*w*). Figure 1 displays the gelatinization curves, while Table 2 exhibits gelatinization parameters including peak viscosity (PV), trough viscosity (TV), breakdown viscosity (BV), final viscosity (FV), setback viscosity (SV), and pasting temperature (PT). As depicted in Figure 1, initially, the viscosities of all samples were nearly identical. Furthermore, the viscosity curves of RS and CP–RS gels all increased as the temperature rose. The distinction observed between RS and CP–RS gels indicated that the primary interactions between the RS and CP–RS mainly occurred throughout the gelatinization phase. 

The PV, TV, and FV of RS tended to decrease when the CP was added, and this effect became increasingly pronounced with the increase in the concentration of CP. CP–RS showed lower PV, TV, and FV compared with native RS. It should be highlighted that the 3.0% CP addition ratio resulted in the lowest PV (2365.67 mPa·s), TV (1821.00 mPa·s), and FV (2752.67 mPa·s), signifying the significant influence on pasting properties due to CP concentration. These findings were same as those by L. Chen et al. [36], who explained that the interaction between amylose and NSPs probably primarily caused the alteration in viscosity. NSPs suppressed starch granule swelling, and this was also recognized as the reason for reduced viscosity [36]. Additional NSPs, like guar and xanthan gums, pullulan, corn fiber gum, and Laminaria japonica polysaccharides decreased PV, TV, and FV through competing with starch granules for moisture [37]. However, the study by J. Zheng et al. [38] found that NSPs affected RS gelatinization depending on NSP level and type, and showed that high-methoxyl pectin, carboxymethyl cellulose, xanthan gum, and konjac glucomannan all increased the PV, TV, and FV of RS, different to CP’s impact on starch gel viscosity observed in the present work.

BV was numerically equivalent to the difference in PV compared with TV, indicating the extent of starch granule disintegration. Generally, a paste with superior stability corresponds to a reduced BV [39]. The BV of native RS was 739.00 mPa∙s. However, gel BV gradually declined after adding CP, decreasing from 585.00 to 544.67 mPa∙s when the CP ratio increased from 0.6% to 3.0%. The reduction in BV was probably associated with progressive coverage of starch granules via CP as the concentration increased, thereby enhancing the stability of the mixtures.

SV can be calculated by subtracting TV from FV, representing reassociation of swollen starch granules, and this method was adopted for describing short-term starch retrogradation [40]. The SV of pure RS was 1284.00 mPa∙s. The SV of CP–RS gels (*p* < 0.05) apparently reduced after adding CP, indicating that CP suppressed gelatinized starch retrogradation. PT denoted the initial temperature of starch granule expansion. Adding polysaccharides has previously been suggested to elevate starch PT through competition against starch granules for water, but the PT of the CP–RS gels declined after adding CP (*p* > 0.05), contrary to research results by X. Xu et al. [41]. This was probably associated with the distinctly competitive interactions of CP compared with starch.

### 3.2. Rheological Properties

#### 3.2.1. Dynamic Rheological Properties

**Figure 2:**
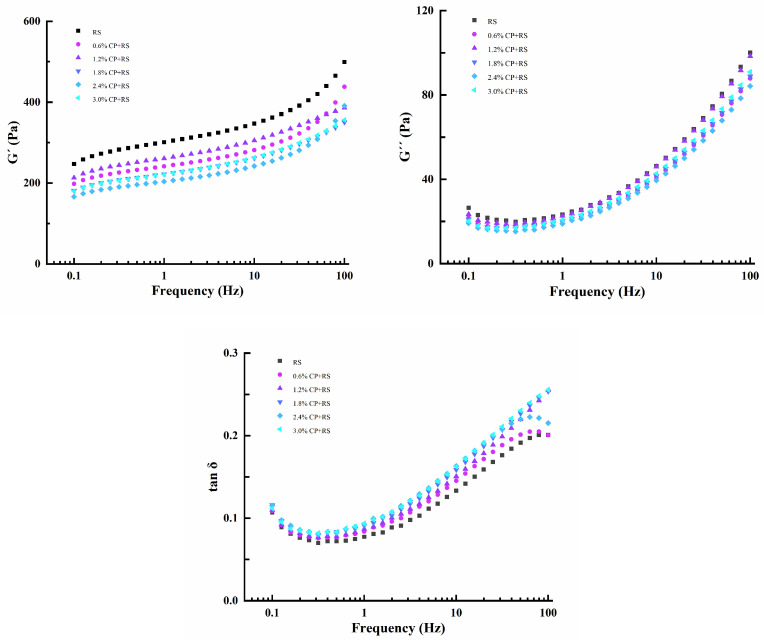
Effect of frequency on the G′, G″ and tan δ of RS and CP–RS gels of diverse CP concentrations at 25 °C.

Figure 2 displays the dynamic rheological measurement analysis results of RS and CP–RS gels. Tan δ value (G″/G′) is an important numerical value used to evaluate viscoelastic behavior. During dynamic rheological assessment, as the frequency was elevated, sample G′ and G″ gradually rose, and G″ decreased consistently compared with G′. This indicated that our samples exhibited characteristics of a typical weak gel [42]. The G′ in each CP–RS gel decreased compared with pure RS, whereas G′ declined after adding CP. Moreover, although the G″ of all CP-RS gels was lower than that of pure starch, the increases were smaller compared with G′. The value of tan δ was less than 1 and increased with the addition of CP. Therefore, it can be inferred that the impact of CP was predominantly on the elasticity of the gelatinized RS. Since the cross-linked network was formed by amylose as the main polymer [43], it is conceivable that the association of CP with leached amylose could have diminished reactivity among the amylose molecules themselves. This reduction in interaction might have resulted in a slower reaggregation of amylose, consequently diminishing the elasticity of the starch paste. 

#### 3.2.2. Static Rheological Measurements

**Figure 3:**
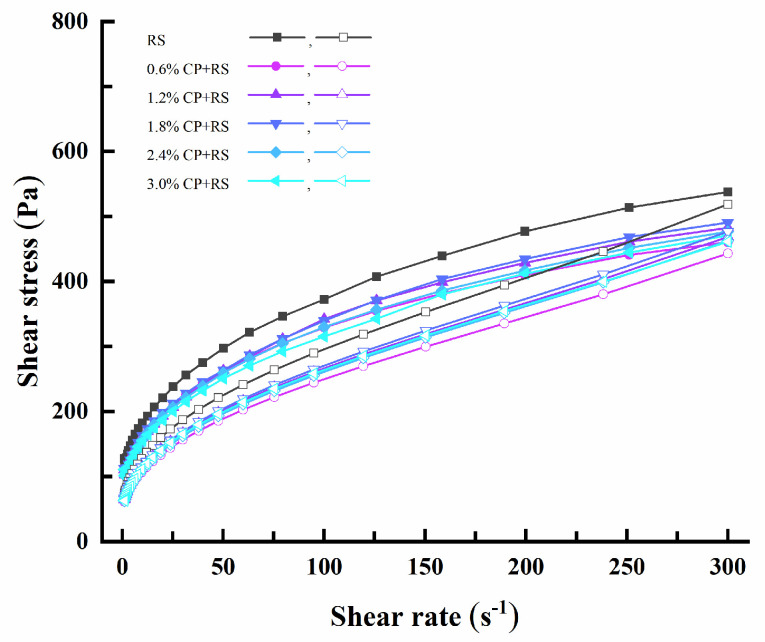
Steady flow curves for RS and CP–RS gels of diverse CP concentrations at 25 °C.

Figure 3 shows the steady shear properties of the CP–RS gels. There was a clockwise thixotropic loop observed between the ascending and descending curves, with the loop area representing the energy necessary for breaking the system structure. In the shear rate range examined in the present research, the Herschel–Bulkley model showed a high fitting degree with the flow curves (R^2^ > 0.99, Table 3). The τ_0_ of the complex system decreased with the addition of CP and the *n* was <1, suggested a decrease in apparent viscosity with the increasing shear rate. Therefore, this was the characteristic starch gel system indicative of a pseudoplastic fluid behavior; similar results have been published for other starch–NSP gels [44]. 

Consistency coefficient (K) accounts for a factor indicating variations in apparent viscosity. An elevated K value corresponds to an increased viscosity. The K values of both the ascending and descending curves in the complex system decreased with the addition of CP, conforming to the observation that CP reduced the viscosity of RS (Table 2); an opposite trend in the K value of RS with added guar gum and locust bean gum was previously reported [41]. These findings suggested that incorporating CP could alter the steady rheological properties of the RS, hinting at a potential association of the starch molecules with CP. A hysteresis loop area (S) contributed to characterizing the degree of gel thixotropy. A larger area indicated a greater degree of external damage to the sample, and also more difficultly returning to its original state. The S of the system declined after adding CP; different from the control, the S of RS gels containing 3.0% CP was reduced by 33.03%. As a result, RS thixotropy was reduced and the RS was less sensitive to shear forces. In the food production process, a smaller thixotropic loop area is desirable, which helps in maintaining the shape of the food and facilitates its processing and manufacturing. The thixotropy of RS can be improved by the addition of CP; thus, different mass ratios of the CP–RS complex system can be used in food applications depending on the specific circumstances.

### 3.3. Thermal Properties

Gelatinization parameters of CP-RS gels were measured through DSC, as shown in Table 4. Temperatures at which gelatinization occurred (To, Tp, and Tc) were typically associated with factors like amylose quality, amylopectin molecule arrangement, and amylopectin double-helical structure generation within crystalline areas of starch granules [11]. CP at low concentrations (0.6–1.2%) made no difference to RS To, Tp, or Tc, while adding 1.8% CP increased them significantly (*p* < 0.05). The pronounced rise of To in the CP–RS gels indicated that the starch granules began to melt at a comparatively elevated temperature [45]. Such findings were consistent with the effects of NSPs on wheat starch and RS [46], which might be understood as indicating NSPs’ ability to immobilize water molecules competing against starch granules for accessible water in gelatinization. This competition reduced the water-to-starch ratio, leading to the increased gelatinization temperature for the CP–RS gels [11]. 

∆H reflects the hydration extent and starch granule disintegration. The gelatinization enthalpy of RS was gradually reduced upon the incorporation of CP, which implied that the addition of CP could reduce the energy necessary for transiting from a suspended state into a gel state. Such findings conform to prior research on the association of native starch with polysaccharide complexes [47]. The ΔHr changes in RS and CP–RS gels during 7 days of storage at 4 °C were determined. The extent of aging in starch pastes was mirrored by their retrogradation rate and enthalpy values. An increase in the retrogradation rate and enthalpy correlated with a more pronounced aging level of starch pastes. The retrogradation rate and the ∆Hr of RS were delayed with the presence of CP and showed a direct correlation with the ratio of CP. With the prolonging storage time, the retrogradation rate and ∆Hr of RS increased significantly from 13.38% (1 d) to 34.89% (7 d) and 1.56 J/g (1 d) to 4.07 J/g (7 d), respectively. Meanwhile, the retrogradation rate and ∆Hr of 3.0% CP–RS increased from only 4.23% (1 d) to 20.33% (7 d) and 0.42 J/g (1 d) to 2.02 J/g (7 ds), separately, implying that CP might suppress long-term RS retrogradation. Such results are consistent with the previous studies involving addition xanthan into RS [47].

### 3.4. SEM Analysis

**Figure 4:**
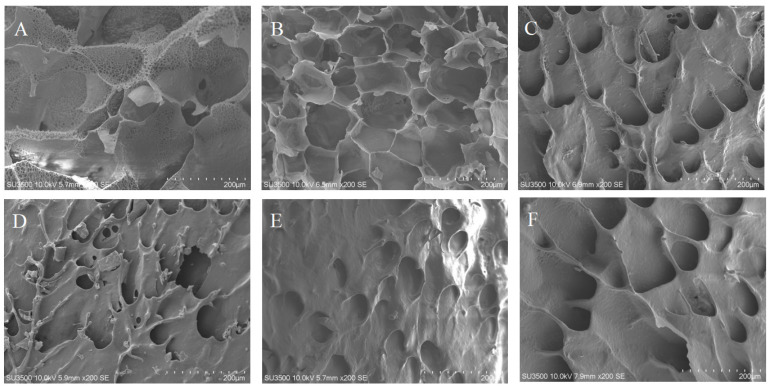
SEM images showing CP–RS gels with diverse CP concentrations of 0.0% (**A**), 0.6% (**B**), 1.2% (**C**), 1.8% (**D**), 2.4% (**E**), 3.0% (**F**).

Figure 4 shows freeze-dried CP–RS gel microstructures (0%, 0.6%, 1.2%, 1.8%, 2.4% and 3.0%, *w*/*w*) under magnification (200×). In all the figures, starch granule structures were not observed, which indicated that the starch had completely gelatinized. RS gel exhibited a disordered network structure that had thin pore walls and irregular pore size, suggesting poor elasticity and low gel hardness. Pore generation inside the gel was due to water sublimation that occurred throughout the freeze drying [48]. However, the low-ratio CP (0.6%) addition demonstrated loosely regular small pores and a honeycomb-like structure. Compared with the control group, as the CP ratio continued to increase (1.2~3.0%), the microstructures of the CP–RS gels exhibited significant differences, the three-dimensional honeycomb structure disappeared, and the structure became very small and dense, presenting as a continuous, lamellar, combined aggregate, similar to cell walls with a smooth surface [49]. This finding was similar to that described by Charoenrein et al. [50] who reported that NSP–RS gels had smaller pores than RS. The starch paste network structure was probably affected by adding CP. Thus, we posited that the alterations in the viscoelastic properties of starch pastes were associated with the CP-induced modifications in the conformation and compactness of their network structure. 

### 3.5. XRD

**Figure 5:**
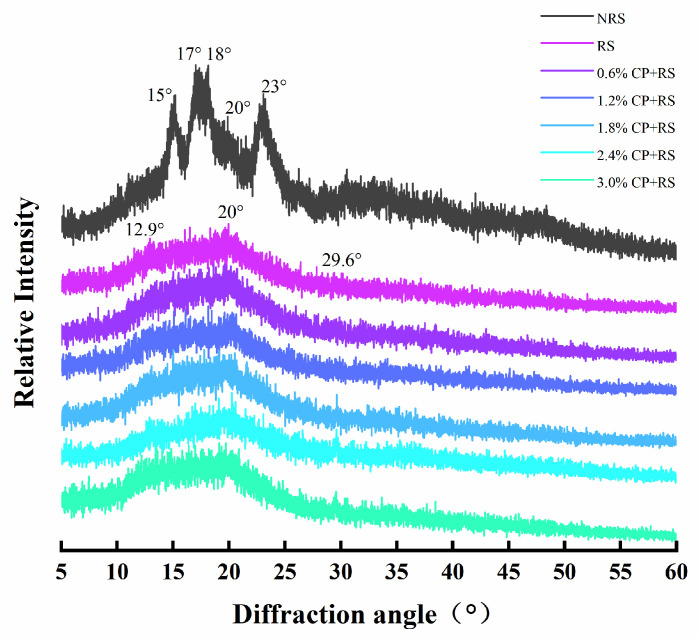
XRD spectra for RS and CP–RS gels of diverse CP concentrations.

XRD patterns for native RS and CP–RS gels are shown in Figure 5. The pure RS displayed a classic “A”-type diffraction pattern in which one potent doublet peak was observed at around an 17° and 18° diffraction angle. It also exhibited stronger single diffraction peaks at 15° and 23°, respectively, close to the results of Ren et al.’s study [33]. The A-type XRD pattern of RS was lost after gelatinization. After gelatinization, the peaks were distributed at 12.9°, 20°, and 29.6°, similar to the study by Q. Zhao et al. [51]. The diffraction pattern changed because the crystalline structure was destroyed because of gelatinization. Nonetheless, the peak shapes of samples across nearly all concentrations displayed no notable variations, indicating that the influence of CP on the crystalline structure of RS might not have been reliant on concentration, nor was a direct correlation apparent. 

### 3.6. FT-IR

**Figure 6:**
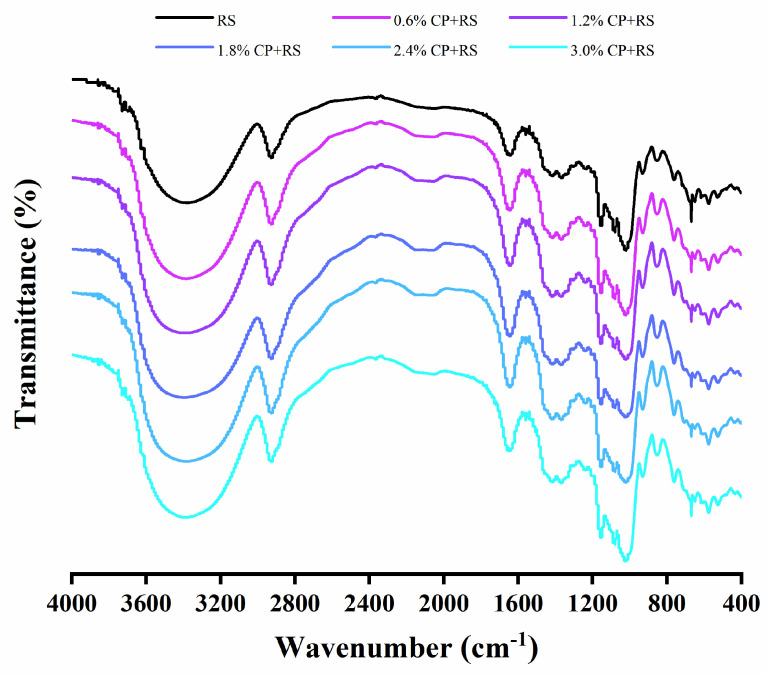
FTIR spectra for RS and CP–RS gels of diverse CP concentrations.

The FT-IR analysis of RS and CP–RS gels is shown in Figure 6. Given that both the starch and CP were polysaccharides with a high hydroxyl group content, an absorption peak was observed close to 3400 cm^−1^, which was associated with O-H stretching vibrations [52]. Typically, the signal seen near 2900 cm^−1^ corresponded to anti-symmetric and symmetric stretching vibrations of -CH and -CH2 groups. Additionally, peaks observed at 1600–1700 cm^−1^ could have been because amide or water were present [53]. In contrast to the pure RS, the introduction of CP into the starch did not result in any new absorption peaks, which suggested the absence of covalent bonding between CP and RS.

### 3.7. In Vitro Digestibility

The starch digestion rate is intimately connected to fluctuations in blood glucose and insulin levels following a meal. Table 5 exhibits the in vitro digestion parameters for CP–RS, including RDS, SDS, and resistant starch. RDS diminished progressively as more CP was incorporated, indicating that CP had the capacity to lessen the digestibility of RS. SDS and resistant starch contents in the CP–RS increased by 32.04% and 39.69%, whereas CP content reached 3.0%, which was significantly different to that of RS (*p* < 0.05). In vitro digestion findings conformed to the sample SEM analysis. The enhanced composite gel microstructure, probably due to the intermolecular interactions among the biomacromolecules, may have contributed to the decreased digestibility of CP–RS with the increased addition of CP. The samples’ compactness was related to digestion appearance, similar to studies on RS–letinous edodes polysaccharide [54]. The reason might have been that CP blocked the effects of digestive enzymes and starch granules. 

### 3.8. Freeze–Thaw Stability

**Figure 7:**
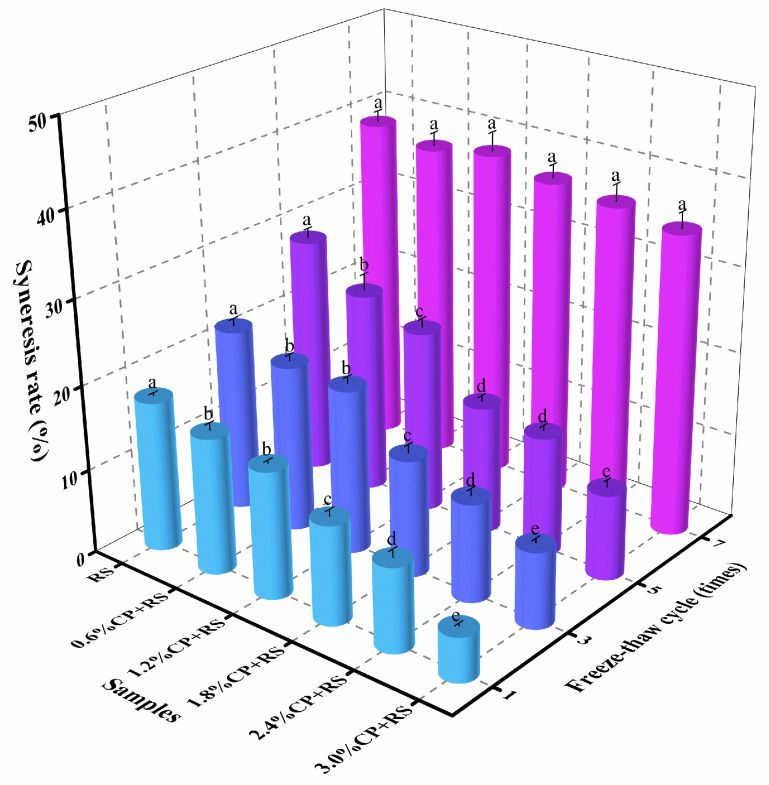
Freeze–thaw stability of RS and CP–RS gels with different CP concentrations. Different letters indicate significant differences among group (*p* < 0.05).

Freeze–thaw stability accounts for a crucial indicator of food quality, and is utilized for assessing starch’s ability to withstand adverse physical alterations induced by exposure to freeze–thaw cycles [55]. The CP–RS syneresis rate over seven freeze–thaw cycles is shown in Figure 7. Compared with pure RS, the syneresis level (17.78%) increased following the initial freeze–thaw cycle. After five freeze–thaw cycles, the syneresis of RS increased from 17.78% to 28.23%. The syneresis of the CP–RS blends with 0.6%, 1.2%, 1.8%, 2.4%, and 3.0% CP content significantly increased from 16.16% to 23.85%, from 15.04% to 21.61%, from 11.73% to 15.11%, from 9.95% to 14.10%, and from 5.26% to 10.07%, respectively. As the number of freeze–thaw cycles increased, so did the gel syneresis values. Throughout multiple cycles, the starch molecules tended to reassemble, aggregate, and potentially form a porous structure, which resulted in water exudation out of the gel matrix, resulting in dehydration and condensation [56,57]. The proof was evident from the reduced syneresis values in the CP–RS gels compared to the control, indicating superior freeze–thaw stability in gels enriched with CP. This could be understood by the fact that the hydrocolloids present within the starch gel bound to water molecules, thus diminishing the syneresis degree across the freeze–thaw cycles [58]. This result was similar to the studies by X. Xu et al. [41]. In summary, the findings suggested that CP has the capacity to prevent dehydration in starch gels throughout freeze–thaw cycles, which could enhance starch-based food product quality.

## 4. Conclusions

This study analyzed how CP affected RS gel’s gelatinization properties, rheological properties, thermal properties, structural properties, digestibility, and freeze–thaw stability. Based on the RVA analysis, as the CP concentration increased, the pasting parameters decreased, indicating that short-term RS retrogradation was inhibited after adding CP. Based on the rheological analysis, RS and CP–RS gels represented pseudoplastic fluids, while the G′ and G″ decreased as the level of CP was elevated. According to the DSC results, adding CP significantly elevated the starch gelatinization temperature while reducing gelatinization enthalpy, and retrogradation enthalpy was obviously reduced during the storage period. SEM analysis revealed that RS surface morphology was adjusted via the addition of CP; the microstructure of the CP–RS composite material became small and dense and presented a continuous lamellar arrangement combined with aggregation, similar to cell walls with a smooth surface. XRD analysis revealed that the crystallization type changed. FTIR analyses revealed that CP was able to interact with RS, but there was no covalent binding between CP and RS. The RS hydrolysis rate was reduced after reducing the RDS level while elevating SDS and resistant starch levels with the addition of CP; all these phenomena could be credited to the reality that during the gelatinization of RS, CP interacted with amylose that was leached out, forming hydrogen bonds. This interaction resulted in the CP enveloping the starch granule surface, thereby hindering starch swelling and pasting. Moreover, the RS gel had markedly enhanced freeze–thaw stability after adding CP. Our results in the present work can help in understanding the interaction between CP and starch.

## Figures and Tables

**Table 1:** The formulations of CP–RS composites.

Smaple	RS (g)	CP (g)
0.6% CP–RS	100	0.6
1.2% CP–RS	100	1.2
1.8% CP–RS	100	1.8
2.4% CP–RS	100	2.4
3.0% CP–RS	100	3.0

**Table 2:** Pasting properties of RS and CP–RS gels of diverse CP concentrations.

Samples	PV (mPa·s)	TV (mPa·s)	BV (mPa·s)	FV (mPa·s)	SV (mPa·s)	PT (°C)
RS	2774.33 ± 69.06 ^a^	2035.33 ± 25.93 ^a^	739.00 ± 77.93 ^a^	3319.33 ± 59.48 ^a^	1284.00 ± 65.09 ^a^	90.03 ± 0.03 ^a^
0.6% CP + RS	2620.00 ± 33.15 ^b^	2035.00 ± 38.16 ^a^	585.00 ± 13.53 ^b^	3087.00 ± 26.91 ^b^	1052.00 ± 15.87 ^b^	89.78 ± 0.38 ^a^
1.2% CP + RS	2507.67 ± 10.02 ^c^	1957.67 ± 11.06 ^b^	550.00 ± 17.35 ^b^	2975.67 ± 9.61 ^c^	1018.00 ± 10.82 ^b^	89.68 ± 0.42 ^a^
1.8% CP + RS	2429.00 ± 51.96 ^cd^	1864.67 ± 5.13 ^c^	564.33 ± 53.35 ^b^	2873.67 ± 37.75 ^d^	1009.00 ± 42.04 ^b^	89.67 ± 0.49 ^a^
2.4% CP + RS	2421.33 ± 19.43 ^cd^	1812.67 ± 42.00 ^c^	608.67 ± 23.46 ^b^	2798.33 ± 24.79 ^de^	985.67 ± 32.47 ^bc^	89.35 ± 0.48 ^a^
3.0% CP + RS	2365.67 ± 68.04 ^d^	1821.00 ± 45.21 ^c^	544.67 ± 34.43 ^b^	2752.67 ± 69.62 ^e^	931.67 ± 28.29 ^c^	89.12 ± 0.83 ^a^

Data represent mean ± SD from triplicates. Diverse lowercase letters in identical columns indicate significant difference (*p* < 0.05).

**Table 3:** Rheological parameters for RS and CP–RS gels of diverse CP concentrations.

Samples	Upward Curve				Downward Curve				S (Pa/s)
	τ_0_ (Pa)	K (Pa·s^n^)	n	R^2^	τ_0_ (Pa)	K (Pa·s^n^)	n	R^2^	
RS	71.58 ± 4.13 ^a^	43.26 ± 2.59 ^a^	0.421 ± 0.01 ^b^	0.999	58.28 ± 3.48 ^a^	19.08 ± 1.59 ^a^	0.552 ± 0.014 ^a^	0.998	19,480
0.6% CP + RS	52.21 ± 5.57 ^c^	44.59 ± 3.7 ^a^	0.392 ± 0.013 ^c^	0.998	49.07 ± 2.8 ^b^	15.23 ± 1.23 ^b^	0.564 ± 0.014 ^a^	0.998	18,732
1.2% CP + RS	55.73 ± 5.56 ^bc^	40.24 ± 3.49 ^a^	0.419 ± 0.014 ^b^	0.998	50.69 ± 2.85 ^b^	17.1 ± 1.29 ^ab^	0.555 ± 0.013 ^a^	0.998	17,159
1.8% CP + RS	57.75 ± 4.65 ^bc^	39.82 ± 2.9 ^a^	0.422 ± 0.012 ^b^	0.998	51.33 ± 2.84 ^b^	17.2 ± 1.28 ^ab^	0.557 ± 0.013 ^a^	0.998	16,783
2.4% CP + RS	54.71 ± 4.43 ^c^	39.76 ± 2.8 ^a^	0.417 ± 0.011 ^b^	0.999	49.6 ± 2.63 ^b^	16.06 ± 1.16 ^b^	0.564 ± 0.012 ^a^	0.999	16,030
3.0% CP + RS	63.94 ± 3.6 ^ab^	32.42 ± 2.13 ^b^	0.446 ± 0.011 ^a^	0.999	47.9 ± 2.57 ^b^	17.7 ± 1.19 ^ab^	0.547 ± 0.011 ^a^	0.999	13,045

τ_0_, yield stress; K, consistency index; n, flow behavior index; R^2^, determination coefficient; S, thixotropic ring area. Diverse lowercase letters in identical columns indicate significant difference *p* < 0.05.

**Table 4:** Thermal transition parameters for RS and CP–RS gels of diverse CP concentrations.

Samples	Gelatinization				Retrogradation			
	To (°C)	Tp (°C)	Tc (°C)	ΔH_0_ (J/g)	R_1_(%)/ΔHr_1_ (J/g)	R_3_(%)/ΔHr_3_ (J/g)	R_5_(%)/ΔHr_5_ (J/g)	R_7_/(%)ΔHr_7_ (J/g)
RS	63.62 ± 0.15 ^c^	67.87 ± 0.05 ^c^	74.29 ± 0.10 ^b^	11.66 ± 0.09 ^a^	13.38 ± 0.02 ^b^/1.56 ± 0.01 ^a^	17.75 ± 0.81 ^a^/2.07 ± 0.11 ^a^	24.78 ± 1.95 ^a^/2.89 ± 0.25 ^a^	34.89 ± 2.30 ^a^/4.07 ± 0.30 ^a^
0.6% CP + RS	63.65 ± 0.06 ^c^	67.94 ± 0.19 ^bc^	74.73 ± 0.34 ^b^	10.95 ± 0.18 ^b^	14.26 ± 0.04 ^a^/1.51 ± 0.03 ^a^	17.38 ± 0.01 ^a^/1.84 ± 0.03 ^b^	21.05 ± 0.40 ^b^/2.23 ± 0.08 ^b^	32.57 ± 0.86 ^b^/3.45 ± 0.15 ^b^
1.2% CP + RS	63.72 ± 0.11 ^c^	67.96 ± 0.31 ^bc^	74.73 ± 0.25 ^b^	10.94 ± 0.24 ^b^	12.61 ± 0.36 ^c^/1.38 ± 0.07 ^b^	16.54 ± 0.00 ^b^/1.81 ± 0.04 ^b^	19.20 ± 0.24 ^c^/2.10 ± 0.02 ^b^	28.33 ± 0.11 ^c^/3.10 ± 0.08 ^c^
1.8% CP + RS	63.99 ± 0.01 ^b^	68.33 ± 0.22 ^ab^	75.38 ± 0.54 ^a^	10.31 ± 0.06 ^c^	10.67 ± 0.13 ^d^/1.10 ± 0.02 ^c^	13.48 ± 0.21 ^c^/1.39 ± 0.03 ^c^	17.26 ± 0.00 ^d^/1.78 ± 0.01 ^c^	28.71 ± 0.71 ^c^/2.96 ± 0.09 ^c^
2.4% CP + RS	64.16 ± 0.20 ^b^	68.56 ± 0.17 ^a^	75.60 ± 0.37 ^a^	10.30 ± 0.04 ^c^	7.28 ± 0.46 ^e^/0.75 ± 0.05 ^d^	13.11 ± 0.53 ^c^/1.35 ± 0.06 ^c^	15.05 ± 0.52 ^e^/1.55 ± 0.06 ^d^	23.88 ± 0.09 ^d^/2.46 ± 0.00 ^d^
3.0% CP + RS	64.43 ± 0.14 ^a^	68.73 ± 0.33 ^a^	75.86 ± 0.16 ^a^	9.93 ± 0.26 ^d^	4.23 ± 0.01 ^f^/0.42 ± 0.01 ^e^	8.86 ± 0.03 ^d^/0.88 ± 0.02 ^d^	11.88 ± 0.01 ^f^/1.18 ± 0.03 ^e^	20.33 ± 0.88 ^e^/2.02 ± 0.14 ^e^

T_o_: onset temperature; T_p_: peak temperature; T_c_: concluding temperature; ΔH_0_, gelatinization enthalpy; ΔHr_1_, ΔHr_3_, ΔHr_5_, ΔHr_7_: retrogradation enthalpy preserved for 1, 3, 5, and 7 d. R_1_, R_3_, R_5_, R_7_: retrogradation rate preserved for 1, 3, 5, and 7 d. Diverse lowercase letters in identical columns indicate significant differences, *p* < 0.05.

**Table 5:** In vitro digestibility for RS and CP–RS gels with different CP concentrations.

Sample	RDS (%)	SDS (%)	Resistant Starch (%)
RS	38.83 ± 0.85 ^a^	27.03 ± 0.91 ^d^	34.14 ± 0.75 ^b^
0.6% CP + RS	35.41 ± 0.80 ^b^	28.97 ± 0.24 ^c^	35.62 ± 0.43 ^ab^
1.2% CP + RS	32.38 ± 0.48 ^c^	29.77 ± 0.19 ^bc^	37.91 ± 0.78 ^ab^
1.8% CP + RS	29.79 ± 0.02 ^d^	30.61 ± 0.65 ^b^	39.60 ± 0.66 ^ab^
2.4% CP + RS	28.62 ± 0.30 ^e^	31.71 ± 0.77 ^a^	39.67 ± 0.54 ^a^
3.0% CP + RS	28.27 ± 0.26 ^e^	32.04 ± 0.97 ^a^	39.69 ± 0.27 ^a^

Columns with different superscripts are significantly different during different samples (*p* < 0.05).

## Data Availability

The original contributions presented in the study are included in the article/Supplementary Materials, further inquiries can be directed to the corresponding author.

## Supplementary Materials

The following supporting information can be downloaded at: https://www.mdpi.com/article/10.3390/foods13152420/s1, Figure S1: Strain sweep curves of the RS and the CP-RS mixtures; Figure S2: FTIR spectra of CP.
