# Supplementary figures and images for: Effects of Cactus Polysaccharide on Pasting, Rheology, Structural Properties, In Vitro Digestibility, and Freeze–Thaw Stability of Rice Starch
Source: Foods. 2024 Jul 30;13(15):2420. doi: 10.3390/foods13152420 (PMC11311433; doi:10.3390/foods13152420)

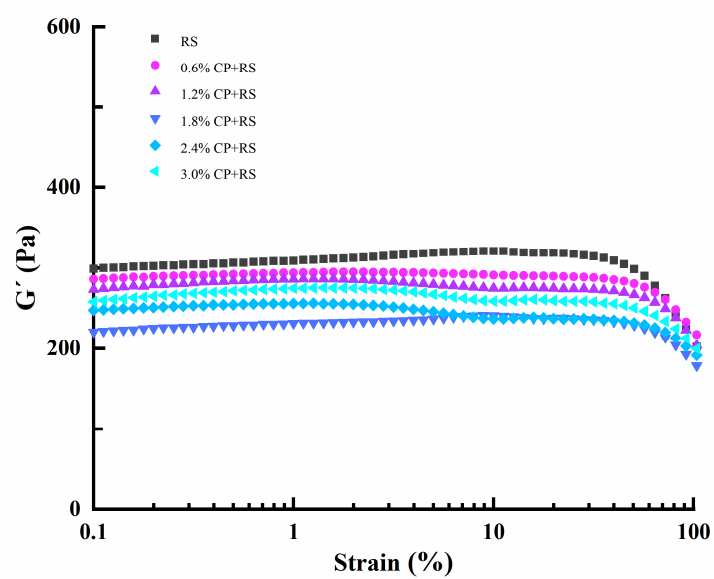

**Figure S1.** Strain sweep curves of the RS and the CP-RS mixtures

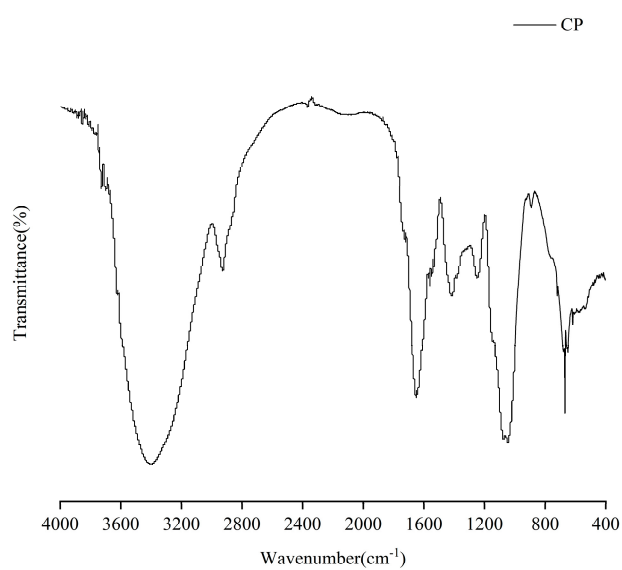

**Figure S2.** FTIR spectra of CP

Supplement: Supplementary file 1 [file foods-13-02420-s001.zip › foods-3078116-supplementary.pdf]
